# Triglyceride-glucose index level and variability and outcomes in patients with acute coronary syndrome undergoing percutaneous coronary intervention: an observational cohort study

**DOI:** 10.1186/s12944-022-01731-w

**Published:** 2022-12-08

**Authors:** Yue Wang, Yue Wang, Shuaifeng Sun, Xinyan Liu, Wenxin Zhao, Wenzheng Li, Min Suo, Zheng Wu, Xiaofan Wu

**Affiliations:** 1 Integrated Ward of Cardiology, Beijing Anzhen Hospital, Capital Medical University, Beijing, 100029 China; 2 Center for Coronary Artery Disease, Beijing Anzhen Hospital, Capital Medical University, Beijing, 100029 China

**Keywords:** Triglyceride-glucose index, Level, Variability, Acute coronary syndrome, Percutaneous coronary intervention

## Abstract

**Background:**

The associations between the long-term triglyceride-glucose (TyG) index level and variability and clinical outcomes in patients with acute coronary syndrome (ACS) undergoing percutaneous coronary intervention (PCI) have not been well studied.

**Methods:**

A total of 1,694 ACS patients with at least three postbaseline TyG index measurements within 2 years after PCI were included in the present study. The TyG index was defined as ln (fasting triglycerides [mg/dL] × fasting plasma glucose [mg/dL]/2). Multivariable-adjusted Cox proportional hazard models were used to examine the association between baseline and mean TyG index levels and TyG index variability and the risk of major adverse cardiovascular and cerebrovascular events (MACCEs).

**Results:**

During the median follow-up of 31 months, the overall incidence of MACCE was 5.9%. Both high baseline and mean TyG index levels were independently associated with an increased risk of MACCEs after adjustment for multiple potential confounders (hazard ratio [HR) 1.76 95% confidence interval [CI] 1.06–2.93; and HR 2.73 95% CI 1.57–4.74). Similarly, higher TyG index variability by successive variation (SD) was well related to a higher prevalence of MACCEs (HR 2.17 95% CI 1.28–3.68). In addition, the mean TyG index level showed a stronger risk prediction for MACCEs than the baseline TyG index level and TyG index-SD (AUCs 0.618 vs 0.566 vs 0.566).

**Conclusions:**

The risk of MACCEs significantly increased with higher baseline and mean TyG index levels, as well as TyG index variability, in patients with ACS undergoing PCI. In particular, the mean TyG index level exhibited the highest predicting ability for MACCEs. Therefore, monitoring the long-term pattern of the TyG index deserves attention in clinical practice.

**Supplementary Information:**

The online version contains supplementary material available at 10.1186/s12944-022-01731-w.

## Background

Acute coronary syndrome (ACS), as the most serious manifestation of coronary artery disease (CAD), remains a leading cause of mortality worldwide [1]. Patients with ACS are still at a heightened risk of cardiovascular events after percutaneous coronary intervention (PCI), despite using current guideline-recommended or evidence-based strategies, such as newer generation drug-eluting stents, optimal antiplatelet therapy (ticagrelor or prasugrel), and intensified lipid-lowering medication [2–4]. Therefore, the identification of residual risk factors for recurrent cardiovascular events is vital to improve clinical management.

Insulin resistance (IR) was reported to be closely associated with ACS onset and poor prognosis [5, 6]. It has been proven that the triglyceride-glucose (TyG) index, calculated as ln [fasting triglycerides (TGs) (mg/dL) × fasting plasma glucose (FPG) (mg/dL)/2], is a reliable and simple surrogate for IR and consistent with the standard measurement of IR [7–9]. Strong correlations have been demonstrated between the TyG index and hypertension, vessel calcification, subclinical CAD, ACS, and stroke [10–15]. Furthermore, it was recently suggested that the TyG index can effectively predict poor outcomes for ACS patients with or without PCI [16, 17]. However, these previous studies only assessed the prediction power of the baseline TyG index and did not determine the association between long-term exposure and variability in the TyG index and adverse cardiovascular outcomes. Whether longitudinal patterns of the TyG index, such as the mean value or visit-to-visit variability, can provide better prognostic information than a single TyG index measurement has not been specifically assessed. Therefore, the present study aimed to investigate the relationships between baseline and mean levels of the TyG index and its variabilities and the incident cardiovascular and cerebrovascular events in ACS patients who underwent PCI and to determine which of these indices was superior for poor prognostication.

## Methods

### Study population

**Fig. 1:**
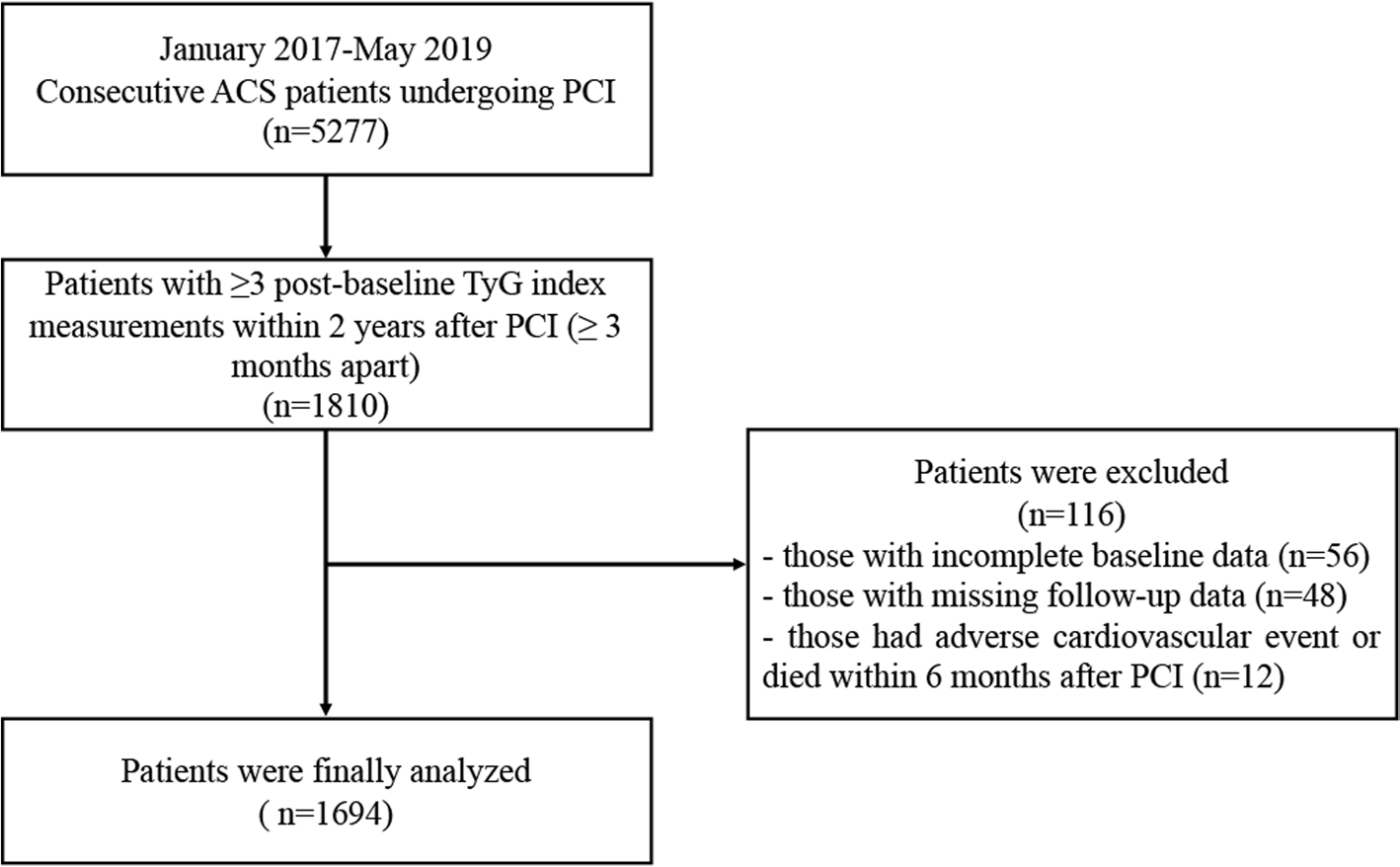
Flow diagram of the study. ACS, acute coronary syndrome; PCI, percutaneous coronary intervention; TyG, triglyceride-glucose

In this single-center retrospective study, a total of 5,277 ACS patients undergoing PCI were assessed from January 2017 to May 2019 at Beijing Anzhen Hospital, Capital Medical University, Beiing, China. Patients lacking at least three postbaseline TyG index measurements within 2 years after PCI (≥ 3 months apart) (*n* = 3467) and those who had adverse cardiovascular events or died within 6 months after PCI (*n* = 12) were excluded. Patients with incomplete baseline data (*n* = 56) and those who missing follow-up data (*n* = 48) were also excluded. Finally, 1,694 participants were included in the present analysis (Fig. 1). All procedures complied with the Declaration of Helsinki and were endorsed by the Ethics Committee and Independent Review Board of Beijing Anzhen Hospital. Informed consent was obtained from the patients before the index PCI.

### Data collection and definition

Patient medical records were reviewed for information on demographics and clinical characteristics, angiographic and procedural details, and laboratory data. After overnight fasting on the day of the coronary procedure, venous blood samples were collected into coagulation-promoting tubes or EDTA anticoagulant tubes and transported on ice to the core laboratory of Beijing Anzhen Hospital within a few hours. Biological markers, including lipid profiles [TG, total cholesterol (TC), low-density lipoprotein cholesterol (LDL-C), high-density lipoprotein cholesterol (HDL-C)], creatinine, uric acid, FPG, and hemoglobin A1c (HbA1c), were analyzed by standard laboratory methods. The coronary angiogram and PCI were performed by experienced interventional cardiologists in accordance with current practice guidelines [18]. All patients received aspirin and ticagrelor for a minimum of 1 year after index PCI.

A previous diagnosis of hypertension, receiving antihypertensive agents, or systolic blood pressure ≥ 140 mmHg and/or diastolic blood pressure ≥ 90 mmHg during the baseline hospitalization were considered hypertensions. A history of diabetes mellitus, receiving glucose-lowering therapy, or HbA1c level ≥ 6.5% during the baseline hospitalization were considered diabetes. A definite diagnosis of dyslipidemia, receiving lipid-lowering agents, LDL-C ≥ 1.8 mmol/L, TG ≥ 2.3 mmol/L, or HDL-C < 1.0 mmol/L during the baseline hospitalization were considered dyslipidemia.

### Assessment of TyG index level and variability

The primary exposure variables were the baseline level, mean level, and variability of the TyG index. The mean TyG index value and TyG index variability were evaluated utilizing fasting TG and FPG measurements beyond 2 months after PCI because fasting TG and FPG levels remained relatively stable after the initial decline. The mean TyG index level was calculated based on the average value across all visits for each participant. TyG index variability was assessed using the intraindividual standard deviation (SD) of TyG index values across visits. Individual measurement numbers ranged as follows: 3 measurements (*n* = 922, 54.4%), 4 measurements (*n* = 343, 20.2%) and ≥ 5 measurements (*n* = 429, 25.4%).

### Follow-up and endpoints

The primary outcome was major adverse cardiovascular and cerebrovascular events (MACCEs), defined as a composite of all-cause death, nonfatal myocardial infarction (MI), unplanned revascularization, and ischemic stroke. The secondary endpoints consisted of the individual components of the primary endpoint. Deaths were considered cardiac unless a definitive noncardiac cause was found [19]. Nonfatal MI was diagnosed using the Fourth Universal Definition of MI [20]. Ischemic stroke was diagnosed as a new neurological deficit with sudden onset caused by ischemic or hemorrhagic events, which lasted at least 24 h or led to death [21]. Unplanned revascularization was defined as any unexpected revascularization of the target or nontarget coronary artery, including PCI or coronary artery bypass grafting (CABG) surgery [19]. Each clinical event was adjudicated by at least two members of the individual clinical event committee. Patients were scheduled for follow-up every 3 months until an endpoint occurred or the follow-up period concluded (31, March 2021).

### Statistical analysis

Continuous variables are expressed as the mean ± SD or medians (interquartile range [IQR]) as appropriate, and comparisons were examined using Student’s t test or the nonparametric Wilcoxon rank-sum test. Categorical variables were summarized as frequencies (percentages) and analyzed with the chi-square test or Fisher’s exact test. Cumulative event curves of the primary endpoint were constructed by the Kaplan–Meier approach, with the log-rank test for the differences among the tertile groups. The associations of three indices of the TyG index with the incident MACCEs were estimated using three multivariable Cox regression models. Hazard ratios (HRs) were reported with 95% confidence intervals (CIs). Model 1 included no adjustments, and Model 2 included adjustments for sex and age. For the baseline TyG index value, Model 3 included additional adjustments for dyslipidemia, diabetes mellitus, hypertension, prior MI, prior PCI, prior CABG, mean stent diameter, β-blocker, oral hypoglycemic agents, insulin, and baseline lipid profiles, and baseline HbA1. For the mean level and variability in the TyG index, Model 3 included additional adjustments for dyslipidemia, diabetes mellitus, hypertension, prior MI, prior PCI, prior CABG, mean stent diameter, β-blockers, oral hypoglycemic agents, insulin, baseline lipid profiles, baseline HbA1c, and baseline TyG index value. Covariates were selected a priori as potential factors with clinical relevance. A trend test in Model 3 using the tertiles as ordinal variables was also performed. Additionally, the predictive performance of the mean level and variability in the TyG index on the secondary endpoints was assessed after adjustment for all the variables in Model 3. The prognostic impact of the mean level and variability in the TyG index on the primary endpoint were further explored in subgroups according to age, sex, body mass index (BMI), LDL-C, and HbA1c. Pairwise comparisons of receiver operating characteristic (ROC) curves were conducted to compare the predictive capabilities of three indices of the TyG index for MACCEs, with differences in the areas under the curves (AUCs) evaluated by Delong’s test. A two-tailed *P* value < 0.05 was considered to indicate statistical significance. All data were analyzed using SPSS 25.0 (IBM Corp., Armonk, NY, USA) and Stata 14.0 (Stata Corp., College Station, TX, USA).

## Results

Of the final 1694 participants, the mean age was 57.8 ± 9.7 years, and 79.0% were male. The mean value of the TyG index during follow-up was 8.73 (IQR 8.42–9.05), and the SD was 0.20 (IQR 0.12–0.29). During the median follow-up of 31 months, 7 (0.4%) all-cause deaths (5 from cardiovascular diseases [CVD]), 17 (1.0%) nonfatal MI, 82 (4.8%) unplanned revascularization, and 5 (0.3%) ischemic strokes occurred. The primary endpoint event, MACCEs, occurred in 100 (5.9%) participants.

### Baseline characteristics

Table 1 summarizes the baseline characteristics according to the occurrence of MACCEs. Patients who experienced MACCEs had higher prevalences of diabetes mellitus, prior MI, prior PCI, and prior CABG; exhibited much higher baseline FPG, baseline HbA1c, mean TyG index value and TyG index–SD; and were more likely to be prescribed glucose-lowering drugs than those who did not experience MACCEs. The baseline characteristics stratified by the mean TyG index value and TyG index–SD are presented in Table S1 and Table S2, respectively.

**Table 1:** Clinical characteristics of the patients stratified by the primary endpoint

	**Total**	**No-MACCE**	**MACCE**	***P* value**
	**(*n* = 1694)**	**(*n* = 1594)**	**(*n* = 100)**	
**Age (y)**	57.8 ± 9.7	57.7 ± 9.7	59 ± 9.8	0.182
**Sex, male**	1339 (79.0)	1261 (79.1)	78 (78.0)	0.792
**BMI, kg/m**^**2**^	26 ± 3.1	26 ± 3.1	26.4 ± 3.3	0.181
**Risk factors, n (%)**				
Hypertension	1025 (60.5)	961 (60.3)	64 (64.0)	0.461
Dyslipidemia	958 (56.6)	895 (56.1)	63 (63.0)	0.180
Diabetes mellitus	618 (36.5)	566 (35.5)	52 (52.0)	0.001
Current smoker	489 (28.9)	453 (28.4)	36 (36.0)	0.105
**Medical history, n (%)**				
Prior MI	229 (13.5)	204 (12.8)	25 (25.0)	0.001
Prior PCI	261 (15.4)	236 (14.8)	25 (25.0)	0.006
Prior CABG	23 (1.4)	18 (1.1)	5 (5.0)	0.001
Prior stroke	104 (6.1)	97 (6.1)	7 (7.0)	0.712
PAD	30 (1.8)	28 (1.8)	2 (2.0)	1.000
CKD	17 (1.0)	16 (1.0)	1 (1.0)	1.000
**ACS type, n (%)**				0.954
STEMI	327 (19.3)	309 (19.4)	18 (18.0)	
NSTEMI	253 (14.9)	237 (14.9)	16 (16.0)	
Unstable angina	1114 (65.8)	1049 (65.8)	65 (65.0)	
**Procedure characteristics**				
Lesion vessel number	2.0 (1.0, 3.0)	2.0 (1.0, 3.0)	2.0 (1.0, 3.0)	0.139
Lesion complexity, n (%)				
Left main lesion	141 (8.3)	137 (8.6)	4 (4.0)	0.107
Bifurcation lesion	181 (10.7)	169 (10.6)	12 (12.0)	0.661
CTO	289 (17.1)	268 (16.8)	21 (21.0)	0.280
Target vessel territory, n (%)				
Left main	96 (5.7)	95 (6.0)	1 (1.0)	0.037
LAD	1012 (59.7)	962 (60.3)	50 (50.0)	0.041
LCX	466 (27.5)	435 (27.3)	31 (31.0)	0.420
RCA	564 (33.3)	522 (32.7)	42 (42.0)	0.057
Multivessel intervention, n (%)	384 (22.7)	364 (22.8)	20 (20.0)	0.511
Stent number	1.0 (1.0, 2.0)	1.0 (1.0, 2.0)	1.5 (1.0, 3.0)	0.417
Mean stent diameter, mm	3.0 ± 0.4	3.0 ± 0.4	2.9 ± 0.4	0.026
Total stent length, mm	35.0 (23.0, 58.0)	35.0 (23.0, 58.0)	36.0 (24.0, 63.5)	0.577
**Laboratory results**				
Baseline LDL-C, mg/dL	95.1 (73.9, 122.6)	95.1 (74.3, 122.2)	94.9 (76.8, 125.7)	0.455
Baseline HDL-C, mg/dL	42.1 ± 9.7	42.2 ± 9.8	40.6 ± 9.2	0.114
Baseline TC, mg/dL	166.5 ± 42.9	166.5 ± 43.2	166.7 ± 39.3	0.955
Baseline TG, mg/dL	124.8 (89.4, 176.1)	115.1 (85.9, 161.3)	119.9 (92.9, 163.1)	0.399
Baseline FPG, mg/dL	104.6 (93.8, 124.6)	104.2 (93.6, 124.0)	112.2 (97.7, 152.4)	0.004
Baseline HbA1c, %	6.4 ± 1.3	6.4 ± 1.3	6.7 ± 1.3	0.010
Baseline creatinine, μmol/L	71.7 ± 16.6	71.7 ± 16.6	71.4 ± 15.7	0.865
Baseline uric acid, μmol/L	355.4 ± 86.9	355.6 ± 87.3	352.8 ± 80.1	0.752
Baseline TyG index	8.82 (8.45, 9.24)	8.82 (8.44, 9.23)	8.97 (8.59, 9.42)	0.027
Mean follow-up TyG index	8.73 (8.42, 9.05)	8.72 (8.40, 9.04)	8.96 (8.55, 9.28)	< 0.001
TyG index-SD	0.20 (0.12, 0.29)	0.20 (0.12, 0.29)	0.23 (0.15, 0.32)	0.025
LVEF, %	61.4 ± 7.8	61.5 ± 7.8	60.5 ± 8.0	0.217
**Medications at discharge, n (%)**				
Aspirin	1694 (100.0)	1594 (100.0)	100 (100.0)	NA
Ticagrelor	1694 (100.0)	1594 (100.0)	100 (100.0)	NA
DAPT interruption in 12 months	550 (32.5)	516 (32.4)	34 (34.0)	0.736
Statin	1674 (98.8)	1575 (98.8)	99 (99.0)	1.000
Ezetimibe	290 (17.1)	273 (17.1)	17 (17.0)	0.974
β-receptor blocker	946 (55.8)	879 (55.1)	67 (67.0)	0.021
ACEI/ARB	881 (52.0)	822 (51.6)	59 (59.0)	0.149
Calcium-channel antagonist	430 (25.4)	409 (25.7)	21 (21.0)	0.299
Oral hypoglycemic agents	379 (22.4)	346 (21.7)	33 (33.0)	0.009
Metformin	181 (10.7)	163 (10.2)	18 (18.0)	0.015
Alpha-glucosidase inhibitor	222(13.1)	203 (12.7)	19 (19.0)	0.072
Meglitinide	83 (4.9)	73 (4.6)	10 (10) .0	0.028
Sulfonylurea	96 (5.7)	88 (5.5)	8 (8.0)	0.298
Thiazolidinediones	14 (0.8)	13 (0.8)	1 (1.0)	1.000
DPP-4 inhibitor	9 (0.5)	9 (0.6)	0 (0)	1.000
SGLT-2 inhibitors	5 (0.3)	5 (0.3)	0 (0)	1.000
GLP-1 receptor agonist	3 (0.2)	3 (0.2)	0 (0)	1.000
Insulin	100(5.9)	87 (5.5)	13 (13.0)	0.002

ACEI/ARB, angiotensin converting enzyme inhibitors/angiotensin receptor blockers; BMI, body mass index; CABG, coronary artery bypass grafting; CKD, chronic kidney disease; CTO, chronic total occlusion; DPP-4, dipeptidyl peptidase-4; FPG, fasting plasma glucose; GLP-1, glucagon-like peptide-1; HbA1c, glycosylated hemoglobin; HDL-C, high density lipoprotein cholesterol; LAD, left anterior descending artery; LCX, left circumflex; LDL-C, low density lipoprotein cholesterol; LVEF, left ventricular ejection fraction; MI, myocardial infarction; NSTEMI, no ST-segment elevation myocardial infarction; PAD, peripheral arterial disease; PCI, percutaneous coronary intervention; RCA, right coronary artery; SGLT-2, sodium-glucose cotransporter-2; STEMI, ST-segment elevation myocardial infarction; TC, total cholesterol; TG, triglyceride

### Clinical outcomes

**Fig. 2:**
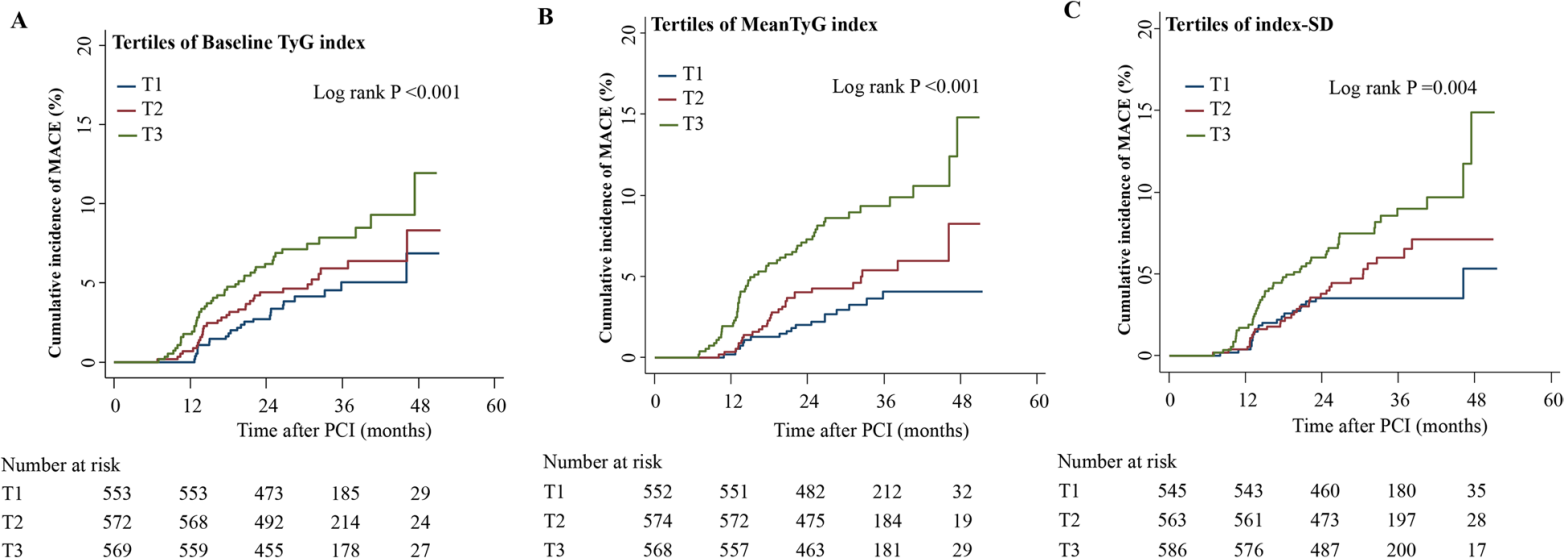
Kaplan–Meier estimation of MACCEs by TyG index level and variability. **A** Cumulative incidences of MACCEs grouped by tertiles of the baseline TyG index. **B** Cumulative incidences of MACCEs grouped by tertiles of the mean TyG index. **C** Cumulative incidences of MACCEs grouped by tertiles of TyG index-SD. MACCEs, major advent cardiovascular events; SD, standard deviation; TyG, triglyceride-glucose

**Fig. 3:**
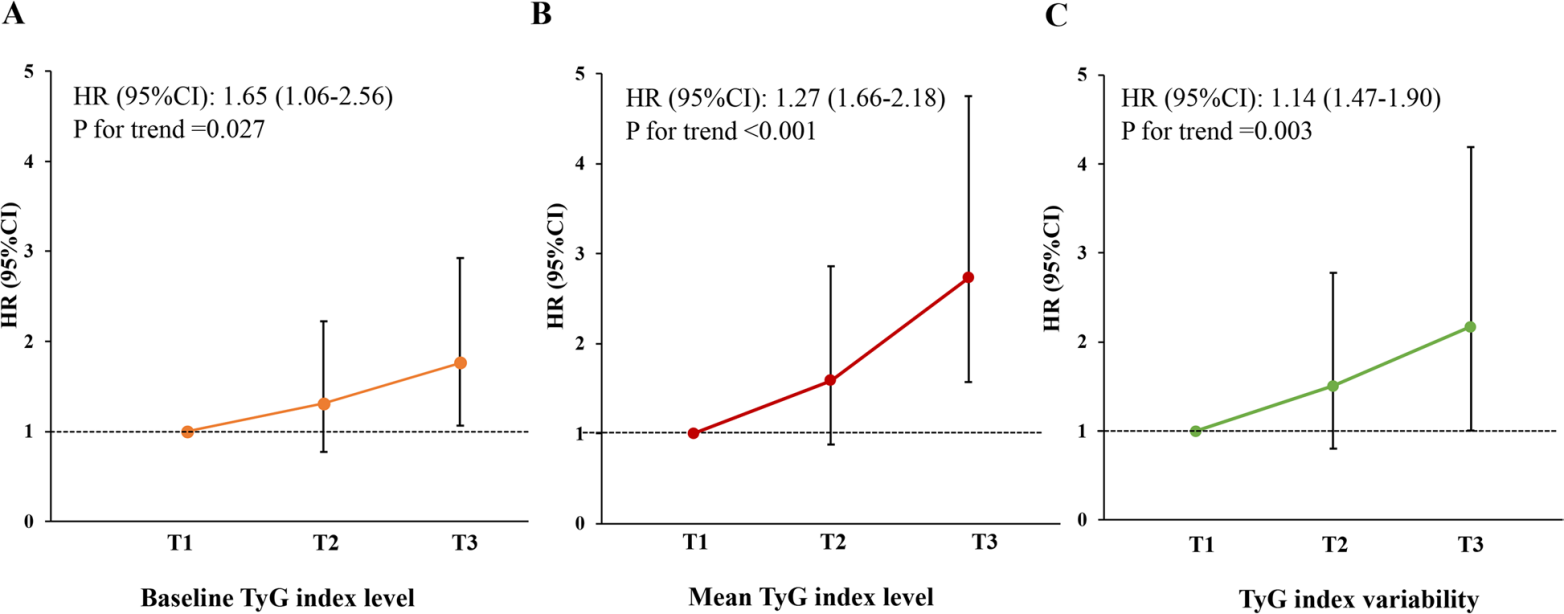
Adjusted hazard ratios for MACCEs by tertiles of baseline level (**A**), mean level (**B**) and variability of TyG index (**C**). The model was adjusted for age, sex, body mass index, hypertension, dyslipidemia, diabetes mellitus, prior MI, prior PCI, prior CABG, mean stent diameter, β-blockers, oral hypoglycemic agents, insulin, baseline LDL-C, baseline TC, baseline HDL-C, and baseline HbA1c for the baseline TyG index and additional adjusted for baseline TG, baseline FPG and the baseline TyG index for the mean TyG index and TyG index variability. CABG, coronary artery bypass grafting; CI, confidence interval; FPG, fasting blood glucose; HbA1c, hemoglobin A1c; HDL-C, high-density lipoprotein cholesterol; HR, hazard ratio; LDL-C, low-density lipoprotein cholesterol; PCI, percutaneous coronary intervention; SD, standard deviation; TC, total cholesterol; TyG, triglyceride-glucose

The log-rank test findings were significant for MACCEs across the tertiles of all indices of the TyG index in the Kaplan–Meier estimate analyses (Fig. 2). Table 2 shows the associations between the three indices of the TyG index and the incidence of MACCEs in the different models. In unadjusted analyses, the rate of MACCEs was significantly higher in the highest baseline TyG index tertile versus the lowest tertile (HR, 1.86; 95% CI 1.13–3.06). After multivariable adjustment (Model 2 or Model 3), differences in MACCE rates remained statistically significant between the highest tertile and the lowest tertile. Similar findings were observed when the mean TyG index value and TyG index–SD were included in these models. After adjustment for variables in Model 3, the highest tertiles of the mean level and variability of the TyG index demonstrated 1.72- and 1.17-fold increased risks of MACCEs versus the lowest tertile, respectively. Moreover, there were stepwise increasing trends in the risk of MACCEs with increasing tertiles of baseline level (*P* = 0.027), mean level (*P* < 0.001), and variability (*P* = 0.003) of the TyG index (Fig. 3).

**Table 2:** Hazard ratios for MACCEs according to the median TyG index level and variability

	**Number**	**Number of events (%)**	**Model 1**		**Model 2**		**Model 3**	
			**HR (95% CI)**	***P* value**	**HR (95% CI)**	***P* value**	**HR (95% CI)**	***P* value**
**Baseline TyG index**								
< 8.59	553	24 (4.3)	Reference	–	Reference	–	Reference	–
8.59–9.08	572	32 (5.6)	1.29 (0.76, 2.18)	0.351	1.34 (0.79, 2.28)	0.279	1.31 (0.77, 2.22)	0.325
≥ 9.08	569	44 (7.7)	1.86 (1.13, 3.06)	0.014	1.97 (1.19, 3.25)	0.008	1.76 (1.06, 2.93)	0.030
**Mean TyG index**								
< 8.53	552	18 (3.3)	Reference	–	Reference	–	Reference	–
8.53–8.93	574	29 (5.1)	1.62 (0.9, 2.92)	0.108	1.64 (0.91, 2.96)	0.098	1.59 (0.88, 2.86)	0.125
≥ 8.93	568	53 (9.3)	3.04 (1.78, 5.18)	< 0.001	3.21 (1.87, 5.51)	< 0.001	2.72 (1.57, 4.74)	< 0.001
**TyG index-SD**								
< 0.15	545	20 (3.7)	Reference	–	Reference	–	Reference	–
0.15–0.26	563	31 (5.5)	1.49 (0.85, 2.62)	0.162	1.51 (0.86, 2.64)	0.153	1.51 (0.86, 2.65)	0.151
≥ 0.26	586	49 (8.4)	2.31 (1.37, 3.88)	0.002	2.39 (1.42, 4.02)	0.001	2.17 (1.28, 3.68)	0.004

CI confidence interval; MACCE, major advent cardiovascular and cerebrovascular event; HR, hazard ratio; SD, standard deviation; TyG, triglyceride glucose

Model 1, unadjusted model; Model 2, adjusted for age and sex; Model 3, adjusted for variables in model 2 plus hypertension, dyslipidemia, diabetes mellitus, prior MI, prior PCI, prior CABG, mean stent diameter, β-blocker, oral hypoglycemic agents, insulin, baseline LDL-C, baseline TC, baseline HDL-C, baseline HbA1C for baseline TyG index; or adjusted for variables in model 2 plus hypertension, dyslipidemia, diabetes mellitus, prior MI, prior PCI, prior CABG, mean stent diameter, β-blocker, oral hypoglycemic agents, insulin, baseline LDL-C, baseline TC, baseline TG, baseline HDL-C, baseline FPG, baseline HbA1C and baseline TyG index for mean TyG index and TyG index-SD

For the secondary endpoints, the risk of unplanned revascularization was significantly higher in the highest baseline TyG index tertile than in the lowest tertile (HR 2.97 95% CI 1.64–5.38; *P* < 0.001), but other cardiovascular events were not statistically significant among these tertiles. Positive associations were observed between the TyG index–SD and the risks of nonfatal MI and unplanned repeat revascularization, but other events did not differ significantly among these tertiles (Table S3).

The impact of TyG index level and variability on the primary outcome were analyzed across subgroups of age, sex, BMI, baseline LDL-C and baseline HbA1c **(**Figure S1 and Figure S2). A significant association between the mean TyG index value or TyG index–SD and MACCE was detected in males or patients with BMI ≤ 25 kg/m^2^ or LDL-C > 70 mg/dL. The risk of MACCEs increased with the mean TyG index level in patients over 65 years, as well as the tertiles of the TyG index–SD in those under 65 years. MACCEs increased substantially with increasing mean level of TyG index regardless of baseline HbA1c, but the positive impact of TyG index variability for MACCEs was not observed in patients with HbA1c ≤ 6.5%. A significant interaction did not exist between both mean value and variability of TyG index and these subgroups.

**Fig. 4:**
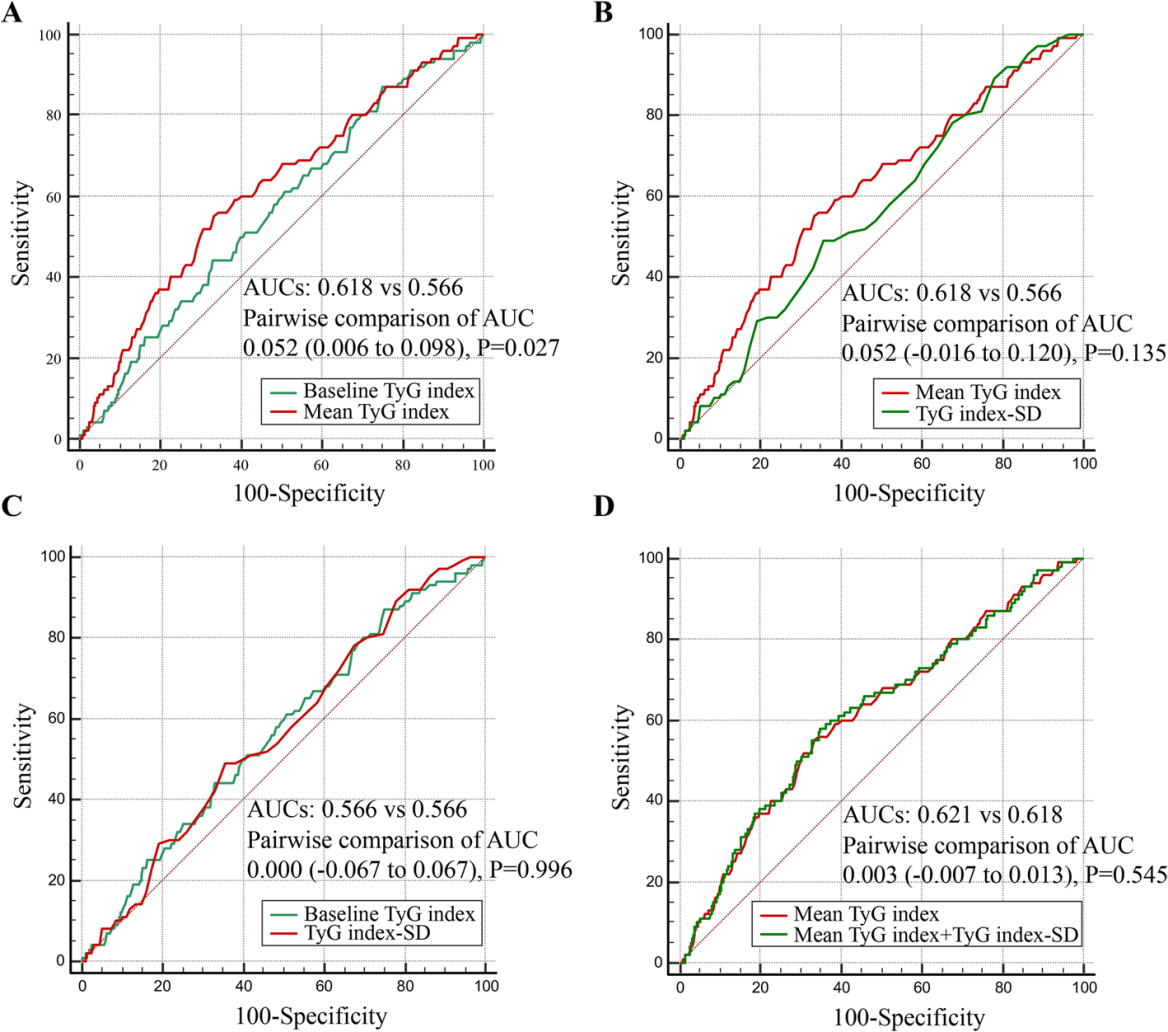
ROC curves of baseline and mean TyG index and TyG index variability related to MACCEs.** A** Baseline TyG index vs mean TyG index;** B** mean TyG index vs TyG index–SD; **C** baseline TyG index vs TyG index–SD; **D** mean TyG index + TyG index–SD vs mean TyG index. CI, confidence interval; HR, hazard ratio; SD, standard deviation; TyG, triglyceride-glucose

ROC curves for three indices of the TyG index related to MACCEs are shown in Fig. 4. The mean TyG index level showed the strongest risk prediction for MACCEs compared with the baseline level of and variability in the TyG index (AUCs 0.618 vs 0.566 vs 0.566). There was no significant difference in AUCs between the baseline level of the TyG index and TyG index–SD (AUCs 0.566 vs 0.566, *P* = 0.996). No significant incremental effect on the prediction of MACCEs after adding the TyG index–SD to the mean TyG index value was observed (AUCs 0.621 vs 0.618, *P* = 0.545).

## Discussion

The present study demonstrated the prognostic roles of baseline and mean TyG index levels and variability in the TyG index in ACS patients undergoing PCI. The major findings were that 1) the incidence of MACCEs increased with increasing baseline or mean level of TyG index even after adjustment for potential confounding factors; 2) similar observations were noted for the relationship of TyG index variability with cardiovascular outcomes; and 3) mean TyG index value showed the most powerful ability to predict risk of MACCEs.

### TyG index level and cardiovascular outcomes

It has been proven that the TyG index has good concordance with the gold standard test for IR and even outperforms the homeostasis model assessment of IR and other alternative indicators (total cholesterol/HDL-C, visceral adiposity index, and apolipoprotein B/apolipoprotein A1) [8, 9, 22]. There has been some evidence that the TyG index is highly related to CVD risk factors and is valuable for the early detection of patients vulnerable to developing CVD [13, 14, 23]. Recently, the baseline TyG index level showed strong predictability for CAD prognosis. A retrospective cohort study on 3,181 patients with acute MI demonstrated that the risk of MACCEs was 19% higher in those with a high TyG index value (HR 1.19 95% CI 1.01–1.41, *P* = 0.046) [24]. Luo et al. [25] observed that the incidence of MACCEs at 1 year after PCI in a STEMI population was 1.53-fold higher in the highest TyG index quartile than in the lowest quartile. Two further cohort studies revealed a strong correlation between an increased TyG index and poor prognosis in ACS patients who underwent PCI and had diabetes mellitus [17, 26]. However, these previous studies only measured the TyG index at baseline. The TyG index is calculated by the fasting FPG and TG levels, both of which vary over time. Consequently, a baseline TyG index measurement does not necessarily reflect that the body state has experienced a high TyG index over long periods of follow-up. Therefore, assessment at multiple time points can characterize the long-term longitudinal pattern of the TyG index and may be more reliable and useful prognostically than a single TyG index measurement. The present study evaluated the impact of the mean TyG index level over time on incident MACCEs in ACS patients with PCI for the first time. The findings provide support for the existing results showing that, in addition to a high baseline TyG index, a high mean level during long-term follow-up can predict future cardiovascular events in the ACS population. Moreover, the mean TyG index showed a better predictive ability than the baseline TyG index, even after adjusting for other conventional risk factors.

Biological plausibility has been suggested for a relationship between the TyG index and MACCEs. First, the TyG index comprehensively reflects the extent of IR, which has been shown to cause endothelial dysfunction, oxidative stress, and the inflammatory response, all of which are important pathogenic factors contributing to the worse prognosis of CVD [27–29]. Second, there was a strong correlation between the TyG index and metabolic disorders, such as higher BMI, LDL-C, TG, and FPG levels, which may contribute to the occurrence of adverse cardiovascular outcomes [26, 30]. Third, several studies have demonstrated the impact of the TyG index on macro- and microvascular damage, arterial stiffness and coronary artery calcification, which have been recognized as major risk factors for CVD [31–34]. Fourth, adiponectin, the most abundant adipocytokine, reduces hepatic gluconeogenesis and increases the utilization of glucose and fatty acids by skeletal muscles, which leads to low TG and FPG levels [35]. Low plasma adiponectin levels have been proposed to be negatively associated with IR and have a crucial role in the pathogenesis of atherosclerosis and ACS [36–38]. These findings may in part explain the association seen between the TyG index and adverse cardiovascular outcomes. More efforts are still required to better interpret the mechanism underlying the finding.

### TyG index variability and cardiovascular outcomes

Recently, visit-to-visit variability in cardiovascular biological measurements, including lipids, glycemic parameters, and blood pressure, has sparked interest as a potential predictor for cardiovascular events [39–41]. A post hoc analysis using data collected in the Treating to New Target (TNT) trial showed that the incidences of any cardiovascular event and coronary event both significantly increased with increasing LDL-C variability in CAD patients (HR 1.11 95% CI 1.07–1.15, *P* < 0.0001 and HR 1.16 95% CI 1.10–1.23, *P* < 0.0001) [39]. A prospective cohort study further suggested that in patients with diabetes, high HbA1c variability predicted a higher rate of in-stent restenosis (HR 3.00 95% CI 1.14–7.92) [40]. A patient-level analysis from seven randomized clinical trials revealed that among patients with CAD, MACCEs were associated with greater blood pressure variability [41]. However, the predictive significance of long-term TyG index variability for cardiovascular outcomes has not been fully clarified. The Kailuan cohort, comprising 62,443 Chinese CVD-free patients, demonstrated that individuals with a higher change in TyG index were more prone to developing CVD [42]. For the first time, the prognostic impact of variability in the TyG index on ACS patients undergoing PCI was investigated in the present study. The results revealed a higher risk of MACCEs with higher TyG index variability, with a 2.73-fold greater risk in the highest tertile than in the lowest tertile. According to these data, less TyG index variability is also important in addition to the TyG index level itself.

The mechanisms linking TyG index variability and MACCEs in ACS patients remain unknown, but there are several potential explanations. First, the TyG index was calculated by TGs and FPG, both of which change over time. It has been shown that glycemic fluctuations increase oxidative stress, inflammatory cytokines, endothelial dysfunction and sympathetic overactivation, and the aforementioned relationships might partly explain the potential correction between TyG index variability and cardiovascular events [43–45]. Second, it is possible that TyG index variability can reflect other pathological conditions associated with increased variability of multiple biological parameters that increase cardiovascular risks. Third, individuals with a higher variability of IR are more likely to suffer from hypertension and diabetes mellitus, all linked to cardiovascular events [11, 46]. An in-depth study of the mechanism behind the relationship is warranted.

Intervention with the TyG index may be beneficial in the long-term management of CAD due to its poor prognostic role in patients with CAD. There is, however, a relative lack of clear evidence in this regard. A previous study showed that the insulin sensitizing agent pioglitazone significantly reduced the incidence of recurrent CVD in patients with diabetes mellitus, partly mediated by increased IR [47]. The new hypoglycemic agent sodium-glucose cotransporter-2 (SGLT-2) inhibitors have been proven to improve poor cardiovascular outcomes, one of the possible reasons being the improvement in IR [48, 49]. A randomized, double-blind trial enrolling 40 patients with prediabetes showed that an 8-week treatment with empagliflozin was able to restore brain insulin sensitivity compared with placebo, which may contribute to the beneficial effects of SGLT-2 inhibitors [50]. Further specific-designed investigation is required to determine whether TyG index medication improves clinical prognosis.

### Comparisons with other studies and what does the current work add to the existing knowledge

Previous studies only explored the impact of the baseline TyG index on worse prognosis in ACS patients with PCI [17, 24–26]. The present study further demonstrated the prognostic value of the mean level and variability of the TyG index for poor cardiovascular outcomes and compared the predictive abilities of the three indicators.

### Study strengths and limitations

The present study has several strengths. For the first time, the study comprehensively investigated the association of three indices of the TyG index (baseline level, mean level, and variability) across visits with clinical outcomes in the ACS population and determined the superiority among these factors for the prediction of poor prognosis. poor prognostic prediction. Several limitations of the study also warrant further consideration. First, since this study was retrospective**,** some residual or unmeasured confounders may not have been excluded. The present findings require confirmation by larger prospective studies. Second, several selection bias may exist because only patients with at least three postbaseline TyG index measurements within 2 years after PCI were included, and the frequency of the measurements varied among the patients. Third, decreased plasma adiponectin levels may be a precursor to future cardiovascular events in ACS patients, but this information was not available in the current database. Fourth, the present results should be cautiously interpreted before generalizing to other racial/ethnic groups as differences in metabolic levels. Fifth, some information, including hypoglycemic therapy or lipid-lowering agents during long-term follow-up, was unavailable, which may have affected the prognostic significance of the TyG index on cardiovascular outcomes.

## Conclusions

In conclusion, high baseline and mean levels of the TyG index, as well as high variability in the TyG index, were independently associated with incident MACCEs in ACS patients undergoing PCI. In particular, the mean TyG index showed the strongest predictive potential for poor prognosis. Thus, in clinical practice, the TyG index can be used as a simple and reliable surrogate marker for IR to provide prognostic information for ACS patients following PCI. Furthermore, monitoring the longitudinal pattern of the TyG index could better identify individuals susceptible to cardiovascular events.

## Supplementary Information

**Additional file 1.**

**Additional file 2.**

**Additional file 3: Table S1.** Clinical characteristics stratified by Mean TyG index level. **Table S2.** Clinical characteristics stratified by TyG index variability **Table S3.** The risk of endpoints based on TyG index level and variability. **Figure S1.** Association of TyG index level and major adverse cardiovascular events across subgroups. Hazard ratios for major adverse cardiovascular events by tertiles of mean TyG index in the overall population (a) and across subgroups of age (b), sex (c), BMI (d), LDL-C (e), and HbA1c (f). BMI, body mass index; HbA1c, hemoglobin A1c; LDL-C, low-density lipoprotein cholesterol. CI, confidence interval; HR, hazard ratio. **Figure S2.** Association of TyG index variability and major adverse cardiovascular events across subgroups. Hazard ratios for major adverse cardiovascular events by tertiles of TyG index-SD in the overall population (a) and across subgroups of age (b), sex (c), BMI (d), LDL-C (e), and HbA1c (f). BMI, body mass index; HbA1c, hemoglobin A1c; LDL-C, low-density lipoprotein cholesterol. CI, confidence interval; HR, hazard ratio; SD, standard deviation.

## Data Availability

The datasets used and/or analyzed during the current study are available from the corresponding author on reasonable request.

## Abbreviations

ACS: Acute coronary syndrome
AUC: Area under the curve
BMI: Body mass index
CABG: Coronary artery bypass grafting
CAD: Coronary artery disease
CIs: Confidence intervals
CVD: Cardiovascular disease
FPG: Fasting plasma glucose
HbA1c: Hemoglobin A1c
HDL-C: High-density lipoprotein cholesterol
HRs: Hazard ratios
IR: Insulin resistance
LDL-C: Low-density lipoprotein cholesterol
MACCEs: Major adverse cardiovascular and cerebrovascular events
MI: Myocardial infarction
PCI: Percutaneous coronary intervention
ROC: Receiver-operating characteristic
SD: Standard deviation
SGLT-2: Sodium-glucose cotransporter 2
TGs: Fasting triglycerides
TyG: Triglyceride glucose
