# Supplementary material for: Triglyceride-glucose index level and variability and outcomes in patients with acute coronary syndrome undergoing percutaneous coronary intervention: an observational cohort study
Source: Lipids Health Dis. 2022 Dec 8;21:134. doi: 10.1186/s12944-022-01731-w (PMC9733246; doi:10.1186/s12944-022-01731-w)
Supplement: Supplementary file 1 — Additional file 1. [file 12944_2022_1731_MOESM1_ESM.pdf]

20220820181416758430790014889984

WORD COUNT 3500

TIME SUBMITTED 20-AUG-2022 06:14AM

PAPER ID 89697728

Background

Acute coronary syndrome (ACS) as the most serious manifestation of coronary artery disease (CAD), remains a leading cause of mortality worldwide [1]. Patients with ACS are still at a heightened risk of cardiovascular events after percutaneous coronary intervention (PCI), despite using current guideline recommended or evidence-based strategies, such as newer generation drug-eluting stents, optimal antiplatelet therapy (ticagrelor or prasugrel), and intensified lipid-lowering medication [2-4]. Therefore, identification of residual risk factors for recurrent cardiovascular events is vital to improve clinical management.

Insulin resistance (IR) was reported to be closely associated with ACS onset and poor prognosis [5,6]. It has been proven that triglyceride-glucose (TyG) index, calculated as ln [fasting triglycerides (TGs) (mg/dL) × fasting plasma glucose (FPG) (mg/dL)/2], is a reliable and simple surrogate for IR and consistent with the standard measurement of IR [7-9]. Strong correlations have been demonstrated between TyG index and hypertension, vessel calcification, and subclinical CAD, ACS, and stroke [10-15]. Furthermore, it is recently suggested that TyG index can predict effectively poor outcomes for ACS patients with or without PCI [16,17]. However, these previous studies only assessed the prediction power of baseline TyG index, and did not determine the association between long-term exposure and variability in TyG index and adverse cardiovascular outcomes. Whether longitudinal patterns of TyG index, such as mean value or visit-to-visit variability, can provide better prognostic information than a single TyG index measurement has not specifically assessed. Therefore, the present study aimed to investigate the relationships between baseline and mean levels of TyG index and its variabilities and the incident cardiovascular and cerebrovascular events in ACS patients who underwent PCI and to determine which of these indices was superior for poor prognostication.

Methods

Study population

In the single-center retrospective study, a total of 5,277 ACS patients undergoing PCI were assessed from January 2017 to May 2019 at Beijing Anzhen Hospital, Capital Medical University, Beiing, China. Patients lacking at least three post-baseline TyG index measurements within 2 years after PCI (≥ 3 months apart) (n=3467), and those who had adverse cardiovascular event or died within 6 months after PCI (n=12) were excluded. Patients with incomplete baseline data (n=56) and those who missing follow-up data (n=48) were also excluded. Finally, 1,694 participants were included in the present analysis **(Figure 1)**. All procedures complied with the Declaration of Helsinki and were endorsed by the Ethics Committee and Independent Review Board of Beijing Anzhen Hospital. Informed consent was obtained from the patients before the index PCI.

Data collection and definition

Patients' medical records were reviewed for information on demographics and clinical characteristics, angiographic and procedural details, and laboratory data. Fasting venous blood samples were collected in the next day after the coronary procedure, and biological markers were analyzed in the core laboratory of Beijing Anzhen Hospital. The coronary angiogram and PCI were performed by experienced interventional cardiologists in accordance with current practice guidelines [18]. All patients received aspirin and ticagrelor for a minimum of 1 years after index PCI.

a previous diagnosis of hypertension, receiving antihypertensive agents, or systolic blood pressure ≥140mmHg and /or diastolic blood pressure ≥90mmHg during the baseline hospitalization, were considered hypertensions. A history of diabetes mellitus, receiving glucose-lowing therapy, or HbA1c level ≥6.5% during the baseline hospitalization were considered diabetes. Definite diagnosis of dyslipidemia, receiving

lipid-lowering agents, or low-density lipoprotein cholesterol (LDL-C) ≥ 1.8 mmol/L, TG ≥ 2.3 mmol/L, or high-density lipoprotein cholesterol (HDL-C) < 1.0 mmol/L during the baseline hospitalization were considered dyslipidemia.

Assessment of TyG index level and variability

The primary exposure variables were baseline level, mean level, and variability of TyG index. Mean TyG index value and TyG index variability were evaluated utilizing fasting TG and FPG measurements beyond 2 months after PCI, because fasting TG and FPG levels remained relatively stable after the initial decline. Mean TyG index level was calculated based on the average value across all visits for each participant. TyG index variability was assessed using the intra-individual standard deviation (SD) of TyG index values across visits. Individual measurement numbers ranged as follows: 3 measurements (n = 922, 54.4%), 4 measurement (n = 343, 20.2%) and ≥5 measurements (n = 429, 25.4%).

Follow-up and endpoints

The primary outcome was major adverse cardiovascular and cerebrovascular events (MACCEs), defined as a composite of all-cause death, nonfatal myocardial infarction (MI), unplanned revascularization, and ischemic stroke. The secondary endpoints consisted of the individual components of the primary endpoint. Deaths were considered cardiac unless a definitive non cardiac cause was found [19]. Nonfatal MI was diagnosed using the Fourth Universal Definition of MI [20]. Ischemic stroke was diagnosed as a new neurological deficit with sudden onset caused by an ischemic or hemorrhagic events, which lasted at least 24 hours or lead to death [21]. Unplanned revascularization was defined as any unexpected revascularization of the target or nontarget coronary artery, including PCI or coronary artery bypass grafting (CABG) surgery [19]. Each clinical event was adjudicated by at least two members of the individual clinical event committee. Patients were scheduled for followed-up every 3 months until an endpoint occurred or the follow-up period

concluded (31, March 2021).

Statistical Analysis

Continuous variables were expressed as mean±SD or medians (interquartile range [IQR]) as appropriate and the comparison were examined using Student's t-test or the nonparametric Wilcoxon test. Categorical variables were summarized as frequency (percentages) and analyzed with Chi-square or Fisher's exact test. cumulative event curves of the primary endpoint were constructed by Kaplan–Meier approach, with log-rank test for the differences among the tertile groups. The associations of three indices of TyG index with the incident MACCEs were estimated using three multivariable Cox regression models. Hazard ratios (HRs) were reported with 95% confidence intervals (CIs). Model 1 included no adjustments and Model 2 included adjustments for sex and age. For baseline TyG index value, Model 3 included additional adjustments for dyslipidemia, diabetes mellitus, hypertension, previous MI, previous PCI, previous CABG, mean stent diameter, β-blocker, oral hypoglycemic agents, insulin, and baseline lipid profiles, and baseline HbA1. For mean level and variability in TyG index, Model 3 included additional adjustments for dyslipidemia, diabetes mellitus, hypertension, previous MI, previous PCI, previous CABG, mean stent diameter, β-blockers, oral hypoglycemic agents, insulin, baseline lipid profiles, baseline HbA1c, and baseline TyG index value. Covariates were selected a priori as potentially as potential factors with clinical relevance. A trend test in Model 3 using the tertiles as ordinal variables was also performed. Additionally, the predictive performance of mean level and variability in TyG index on the secondary endpoints was assessed after adjustment for all the variables in Model 3. The prognostic impact of mean level and variability in TyG index on the primary endpoint were further explored in subgroups according to age, sex, body mass index (BMI), LDL-C, and HbA1c. Pairwise comparisons of receiver-operating characteristic (ROC) curves were conducted to compare the predictive capabilities of three indices of TyG index for MACCEs, with difference in the area under the

curves (AUCs) evaluated by Delong's test. Two-tailed *P* value < 0.05 was considered to indicate statistical significance. All data were analyzed using SPSS 25.0 (IBM Corp., Armonk, NY, USA) and Stata 14.0 (Stata Corp., College Station, TX, USA).

Results

Of the final 1694 participants, the mean age was 57.8±9.7 years and 79.0% were male. The mean value of TyG index during follow-up was 8.73 (IQR 8.42-9.05), and the SD of which was 0.20 (IQR 0.12–0.29). During the median follow-up of 31 months, 7 (0.4%) all-cause death (5 from cardiovascular diseases [CVD]), 17 (1.0%) non-fatal MI, 82 (4.8%) unplanned revascularization, and 5 (0.3%) ischemic strokes occurred. The primary endpoint event MACCEs occurred in 100 (5.9%) participants.

Baseline characteristics

Table 1 summarizes the baseline characteristics according to the occurrence of MACCEs. Patients who experienced MACCEs had higher prevalences of previous MI, previous PCI, previous CABG, and diabetes mellitus, exhibited much higher baseline FPG, baseline HbA1c, mean TyG index value and TyG index–SD; and were more likely to be prescribed glucose-lowering drugs than who did not experience MACCEs. The baseline characteristics stratified by mean TyG index value and TyG index–SD are presented in **Table S1** and **Table S2**, respectively.

Clinical outcomes

The log-rank test findings were significant for MACCEs across the tertiles of all indices of TyG index in the Kaplan–Meier estimate analyses (**Figure 2**). **Table 2** shows the associations between three indices of TyG index and the incidence of MACCEs in the different models. In unadjusted analyses, the rate of MACCEs was significantly higher in the highest baseline TyG index tertile versus the lowest one (HR, 1.86; 95%CI 1.13–3.06). After multivariable adjustment (Model 2 or Model 3), differences in MACCEs rates remained

statistically significant between the highest tertile and the lowest tertile. Similar findings were observed when mean TyG index value and TyG index–SD were included in these models. After adjustment for variables in Model 3, the highest tertiles of mean level and variability of TyG index demonstrated 1.72- and 1.17-fold increased risks of MACCEs versus the lowest tertile, respectively. Moreover, there were stepwise increasing trends in the risk of MACCEs with increasing tertiles of baseline level ($P = 0.027$), mean level ($P < 0.001$), and variability ($P = 0.003$) of TyG index (**Figure 3**)

For the secondary endpoints, the risk of unplanned revascularization was significantly higher in the highest baseline TyG index tertile compared with the lowest tertile (HR 2.97 95% CI 1.64-5.38; $P < 0.001$), but other cardiovascular events were not statistically significant among these tertiles. Positive associations were observed between TyG index–SD and the risks of non-fatal MI and unplanned repeat revascularization, but other events did not differ significantly among these tertiles (**Table S3**).

The impact of TyG index level and variability on the primary outcome were analyzed across subgroups of age, sex, BMI, baseline LDL-C and baseline HbA1c (**Figure S1 and Figure S2**). A significant association between mean TyG index value or TyG index–SD and MACCE was detected in males or patients with BMI≤25kg/m^2 or LDL-C>70mg/dL. The risk of MACCEs increased with mean TyG index level in patients over 65 years, as well as the tertiles of TyG index–SD in those under 65 years. MACCEs increased substantially with increasing mean level of TyG index regardless of baseline HbA1c, but the positive impact of TyG index variability for MACCEs was not observed in patients with HbA1c ≤6.5%. A significant interaction did not exist between both mean value and variability of TyG index and these subgroups.

ROC curves for three indices of TyG index related to MACCE are shown in **Figure 4**. Mean TyG index level showed the strongest risk prediction for MACCE than baseline level of and variability in TyG index (AUCs 0.618 vs 0.566 vs 0.566). There was no significant difference in AUCs between baseline level of

TyG index and TyG index–SD (AUCs 0.566 vs 0.566, $P = 0.996$). No significant incremental effect on the prediction of MACCE after adding TyG index–SD to mean TyG index value was observed (AUCs 0.621 vs 0.618, $P = 0.545$).

Discussion

The present study demonstrated the prognostic roles of baseline and mean TyG index level and variability in TyG index in ACS patients undergoing PCI. The major findings were that 1) the incidence of MACCEs increased with increasing baseline or mean level of TyG index even after adjustment for potential confounding factors; 2) similar observations were noted for the relationship of TyG index variability with cardiovascular outcomes; and 3) mean TyG index value showed the most powerful ability to predict risk of MACCEs.

TyG index level and cardiovascular outcomes

It has been proven that TyG index have a good concordance with the gold standard test for IR, and even outperforms the homeostasis model assessment of IR and other alternative indicators (total cholesterol /HDL-C, visceral adiposity index, and apolipoprotein B/apolipoprotein A1) [8,9,22]. There has been some evidence that TyG index is highly related with CVD risk factors, and is valuable for early detection of patients vulnerable to developing CVD [13,14,23]. Recently, baseline TyG index level showed strong predictability for CAD prognosis. A retrospective cohort study on 3,181 patients with acute MI demonstrated that the risk of MACCEs was 19% higher in those with a high TyG index value (HR1.19 95% CI 1.01-1.41, $P = 0.046$) [24]. Luo et al. [25] observed that the incidence of MACCEs at 1 year after PCI in a STEMI population was 1.53-fold higher in the highest TyG index quartile than the lowest one. Two further cohort studies revealed a strong correlation between increased TyG index and poor prognosis in ACS patients who underwent PCI and had diabetes mellitus [17,26]. However, these previous studies only measured TyG index

at baseline. TyG index is calculated by the fasting FPG and TG levels, both of which vary over time. Consequently, a baseline TyG index measurement does not necessarily reflect the body state has experienced a high TyG index over long periods of follow-up. Therefore, assessment at multiple time points can characterize the long-term longitudinal pattern of TyG index and may be more reliable and useful prognostically than a single TyG index measurement. The present study evaluated the impact of mean TyG index level over time on the incident MACCEs in ACS patients with PCI for the first time. The findings provide support for the existing results showing that, in addition to a high baseline TyG index, a high mean level during long-term follow-up, can predict future cardiovascular events in ACS population. Moreover, mean TyG index showed a better predictive ability than baseline TyG index, even other conventional risk factors being adjusted.

Biological plausibility has been suggested for a relationship between TyG index and MACCEs. First, TyG index reflect comprehensively the extent of IR, which have been shown to cause endothelial dysfunction, oxidative stress, and inflammatory response, all of which are important pathogenic factors contributing to the worse prognosis of CVD [27-29]. Second, there was a strong correlation between TyG index and metabolic disorders, such as higher BMI, LDL-C, TG, and FPG levels, which may contribute to the occurrence of adverse cardiovascular outcomes [26,30]. Third, several studies have demonstrated the impact of TyG index on macro- and microvascular damage, arterial stiffness and coronary artery calcification, which have been recognized major risk factors of CVD [31-34]. More efforts are still required to better interpret the mechanism underlying the finding.

TyG index variability and cardiovascular outcomes

Recently, visit-to-visit variability in cardiovascular biological measurements, including lipids, glycemic parameters, and blood pressure, has sparked interest as potential predictors for cardiovascular

events [35-37]. a post-hoc analysis using data collected in the Treating to New Target (TNT) trial showed that the incidences of any cardiovascular event and coronary event both significantly increased with increasing LDL-C variability in CAD patients (HR 1.11 95% CI 1.07-1.15, $P < 0.0001$ and HR 1.16 95% CI 1.10-1.23, $P < 0.0001$) [35]. A prospective cohort study further suggested that in patients with diabetes, high HbA1c variability predicted a higher rate of in-stent restenosis (HR 3.00 95% CI 1.14-7.92) [36]. A patient-level analysis from seven randomized clinical trials revealed that among patients with CAD, MACCEs were associated with greater blood pressure variability [37]. However, the predictive significance of long-term TyG index variability for cardiovascular outcomes has not been fully clarified. The Kailuan cohort, comprising 62,443 Chinese CVD-free patients, demonstrated that individuals with a higher change in TyG index were more prone to developing CVD [38]. For the first time, the prognostic impact of variability in TyG index on ACS patients undergoing PCI was investigated in the present study. The results revealed a higher risk of MACCEs with higher TyG index variability, with a 2.73-fold greater risk in the highest tertile than the lowest one. According to these data, less TyG index variability is also important in addition to TyG index level itself.

The mechanisms linking TyG index variability and MACCEs in ACS patients remain unknown, but there are several potential explanations. First, TyG index was calculated by TGs and FPG, both of which change over time. It has been shown that glycemic fluctuations increased oxidative stress, inflammatory cytokines, endothelial dysfunction and sympathetic overactivation, the aforementioned relationships might partly explain the potential correction between TyG index variability and cardiovascular events [39-41]. Second, it is possible that TyG index variability can reflect other pathological conditions associated with increased variability of multiple biological parameters that increase cardiovascular risks. Third, individuals with a higher variability of IR are more likely to suffer from hypertension and diabetes mellitus, all linked

to cardiovascular events [42,43]. An in-depth study of the mechanism behind the relationship is warranted.

Taking intervention of TyG index may be beneficial in the long-term management of CAD due to its poor prognostic role in patients with CAD. There is, however, a relative lack of clear evidence in this regard. A previous study showed that the insulin sensitizing agent pioglitazone significantly reduced the incidence of recurrent CVD in patients with diabetes mellitus, partly mediated by increased IR [44]. The new hypoglycemic agents Sodium-glucose cotransporter-2 (SGLT-2) inhibitors have been proven to improve poor cardiovascular outcomes, one of possible reasons being the improvement in IR [45,46]. A randomized, double-blind trial, enrolling 40 patients with prediabetes, showed that an 8-week treatment with empagliflozin was able to restore brain insulin sensitivity compared with placebo, which may contribute to the beneficial effects of SGLT-2 inhibitors [47]. Further specific-designed investigation is required to determine whether TyG index medication improves clinical prognosis.

Comparisons with other studies and what does the current work add to the existing knowledge

Previous studies only explored the impact of baseline TyG index on worse prognosis in ACS patients with PCI. The present study further demonstrated the prognostic value of mean level and variability of TyG index for poor cardiovascular outcomes, and compared the predictive abilities of the three indicators.

Study strengths and limitations

the present study has several strengths. For the first time, the study comprehensively investigated the association of three indices of TyG index (baseline level, mean level, and variability) across visits with clinical outcomes in ACS population, and determined the superiority among these factors for prediction of poor prognosis. poor prognostic prediction. Several limitations of the study also warrant further consideration. First, since this study was retrospective, some residual or unmeasured confounders may not have been excluded. The present findings require confirmation by larger prospective studies. Second, several

selection bias may exist, because only patients with least three postbaseline TyG index measurements within 2 years after PCI were included and the frequency of the measurements varied among the patients. Third, the present results should be cautiously interpreted before generalizing to other racial/ethnic groups as differences in metabolic levels. Fourth, some information including hypoglycemic therapy or lipid-lowering agents during long-term follow-up, was unavailable, which may have affected the prognostic significance of TyG index on cardiovascular outcomes.

Conclusions

In conclusion, high baseline and mean level of TyG index, as well as high variability in TyG index were independently associated with the incident MACCEs s in ACS patients undergoing PCI. In particular, mean TyG index showed the strongest predictive potential for poor prognosis. Thus, in clinical practice, TyG index can be usd as a simple and reliable surrogate marker for IR to provide prognostic information for ACS patients following PCI. Furthermore, monitoring the longitudinal patterns of TyG index could better identify individuals susceptible to cardiovascular events.

20220820181416758430790014889984

ORIGINALITY REPORT

14%

SIMILARITY INDEX

PRIMARY SOURCES

1	Haibin Li, Yingting Zuo, Frank Qian, Shuohua Chen, Xue Tian, Penglian Wang, Xia Li, Xiuhua Guo, Shouling Wu, Anxin Wang. "Triglyceride-glucose index variability and incident cardiovascular disease: a prospective cohort study", Cardiovascular Diabetology, 2022<br>Crossref	56 words — 2%
2	www.researchsquare.com<br>Internet	39 words — 1%
3	Anran Wang, Yapeng Li, Lue Zhou, Kai Liu et al. "Triglyceride-Glucose Index Is Related to Carotid Plaque and Its Stability in Nondiabetic Adults: A Cross-Sectional Study", Frontiers in Neurology, 2022<br>Crossref	26 words — 1%
4	jgc301.com<br>Internet	22 words — 1%
5	Www.hindawi.com<br>Internet	20 words — 1%
6	academic.oup.com<br>Internet	20 words — 1%
7	www.primescholars.com<br>Internet	20 words — 1%

8	www.science.gov<br>Internet	20 words — 1%
9	www.ncbi.nlm.nih.gov<br>Internet	19 words — 1%
10	Qi Zhao, Ting-Yu Zhang, Yu-Jing Cheng, Yue Ma, Ying-Kai Xu, Jia-Qi Yang, Yujie Zhou. "Impacts of triglycerides-glucose index on prognosis of patients with type 2 diabetes mellitus and non-ST-segment elevation acute coronary syndrome: results from an observational cohort study in China", Research Square Platform LLC, 2020<br>Crossref Posted Content	15 words — < 1%
11	Su Zou, Yingjia Xu. "Association of the triglyceride-glucose index and major adverse cardiac and cerebrovascular events in female patients undergoing percutaneous coronary intervention with drug-eluting stents: A retrospective study", Diabetes Research and Clinical Practice, 2021<br>Crossref	14 words — < 1%
12	m.scirp.org<br>Internet	14 words — < 1%
13	Ito, Matthew K, Rebecca J Cheung, Eric K Gupta, Kimberly K Birtcher, Pang H Chong, Theresa M Bianco, and Barry E Bleske. "Key Articles, Guidelines, and Consensus Papers Relative to the Treatment of Dyslipidemias—2005", Pharmacotherapy The Journal of Human Pharmacology and Drug Therapy, 2006.<br>Crossref	13 words — < 1%
14	"Abstracts of 51st EASD Annual Meeting", Diabetologia, 2015<br>Crossref	12 words — < 1%

15 579leader.com
Internet
12 words — < 1%

16 bio-protocol.org
Internet
11 words — < 1%

17 repo.lib.tokushima-u.ac.jp
Internet
11 words — < 1%

18 jeccr.biomedcentral.com
Internet
10 words — < 1%

19 pure.rug.nl
Internet
10 words — < 1%

20 www.journalcra.com
Internet
10 words — < 1%

21 E. Romagnoli. "Euroscore as predictor of in-hospital mortality after percutaneous coronary intervention", Heart, 1/20/2008
Crossref
9 words — < 1%

22 Jieyun You, Liming Gao, Yunli Shen, Wei Guo, Xingxu Wang, Qing Wan, Xiaoyan Wang, Jian Wu, Qi Zhang. "Predictors and long-term prognosis of left ventricular aneurysm in patients with acute anterior myocardial infarction treated with primary percutaneous coronary intervention in the contemporary era", Journal of Thoracic Disease, 2021
Crossref
9 words — < 1%

23 www.jstage.jst.go.jp
Internet
9 words — < 1%

24 Bingjun Zhang, Lingling Liu, Hengfang Ruan, Qiang Zhu, Dafan Yu, Yu Yang, Zhengqi Lu. "Triglyceride-glucose index linked to hospital mortality in critically ill stroke
8 words — < 1%

patients: an observational multicentre study on eICU database", Research Square, 2020
Crossref

25 Yongkang Su, Shuxia Wang, Jin Sun, Yan Zhang et al. "Triglyceride Glucose Index Associated With Arterial Stiffness in Chinese Community-Dwelling Elderly", Frontiers in Cardiovascular Medicine, 2021 — 8 words — < 1%
Crossref

26 Yueqiao Si, Wenjun Fan, Chao Han, Jingyi Liu, Lixian Sun. "Atherogenic Index of Plasma, Triglyceride-Glucose Index and Monocyte-to-Lymphocyte Ratio for Predicting Subclinical Coronary Artery Disease", Research Square Platform LLC, 2020 — 8 words — < 1%
Crossref Posted Content

27 downloads.hindawi.com — 8 words — < 1%
Internet

28 library.unisel.edu.my — 8 words — < 1%
Internet

29 link.springer.com — 8 words — < 1%
Internet

30 mafiadoc.com — 8 words — < 1%
Internet

31 medinform.jmir.org — 8 words — < 1%
Internet

32 raw.githubusercontent.com — 8 words — < 1%
Internet

33 Yue Zhang, Xiaosong Ding, Bing Hua, Qingbo Liu, Hui Gao, Hui Chen, Xue-Qiao Zhao, Weiping Li, — 7 words — < 1%

Hongwei Li. "High triglyceride-glucose index is associated with adverse cardiovascular outcomes in patients with acute myocardial infarction", Research Square Platform LLC, 2020
Crossref Posted Content

34 Min Kyung Kim, Chul Woo Ahn, Shinae Kang, Ji Sun Nam, Kyung Rae Kim, Jong Suk Park. "Relationship between the triglyceride glucose index and coronary artery calcification in Korean adults", Cardiovascular Diabetology, 2017
Crossref
6 words — < 1%

35 Yang, M. S., J. A. Burr, and J. E. Mutchler. "The Prevalence of Sensory Deficits, Functional Limitations, and Disability Among Older Southeast Asians in the United States", Journal of Aging and Health, 2012.
Crossref
6 words — < 1%

EXCLUDE QUOTES	OFF	EXCLUDE SOURCES	OFF
EXCLUDE BIBLIOGRAPHY	ON	EXCLUDE MATCHES	OFF
