# Supplementary material for: Triglyceride-glucose index level and variability and outcomes in patients with acute coronary syndrome undergoing percutaneous coronary intervention: an observational cohort study
Source: Lipids Health Dis. 2022 Dec 8;21:134. doi: 10.1186/s12944-022-01731-w (PMC9733246; doi:10.1186/s12944-022-01731-w)
Supplement: Supplementary file 2 — Additional file 2. [file 12944_2022_1731_MOESM2_ESM.pdf]

edanz 理文编辑
INNOVATIVE SCIENTIFIC SOLUTIONS

Certificate of Editing

Edited provisional title
Triglyceride-glucose index level and variability and outcomes in patient with acute coronary syndrome undergoing percutaneous coronary intervention: an observational cohort study

Client name and institution
Yue Wang,Xiaofan Wu, Beijing Anzhen Hospital, Capital Medical University

Date Completed
2022-08-18

Identification code
110196

Certificate issued by
Koji Yamashita
Managing Director and CEO

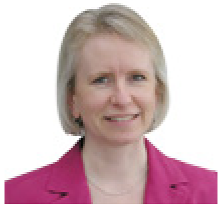

Expert Editor: Alison Sherwin
1992 PhD Biochemistry
University of Kent
Biological Sciences, Medical and Health Sciences , Biochemistry and Cell Biology

www.liwenbianji.cn
While this certificate confirms the authors have used Edanz's editing services, we cannot guarantee that additional changes have not been made after our edits.
