# Supplementary material for: Triglyceride-glucose index level and variability and outcomes in patients with acute coronary syndrome undergoing percutaneous coronary intervention: an observational cohort study
Source: Lipids Health Dis. 2022 Dec 8;21:134. doi: 10.1186/s12944-022-01731-w (PMC9733246; doi:10.1186/s12944-022-01731-w)
Supplement: Supplementary file 3 — Additional file 3: Table S1. Clinical characteristics stratified by Mean TyG index level. Table S2. Clinical characteristics stratified by TyG index variability Table S3. The risk of endpoints based on TyG index level and variability. Figure S1. Association of TyG index level and major adverse cardiovascular events across subgroups. Hazard ratios for major adverse cardiovascular events by tertiles of mean TyG index in the overall population (a) and across subgroups of age (b), sex (c), BMI (d), LDL-C (e), and HbA1c (f). BMI, body mass index; HbA1c, hemoglobin A1c; LDL-C, low-density lipoprotein cholesterol. CI, confidence interval; HR, hazard ratio. Figure S2. Association of TyG index variability and major adverse cardiovascular events across subgroups. Hazard ratios for major adverse cardiovascular events by tertiles of TyG index-SD in the overall population (a) and across subgroups of age (b), sex (c), BMI (d), LDL-C (e), and HbA1c (f). BMI, body mass index; HbA1c, hemoglobin A1c; LDL-C, low-density lipoprotein cholesterol. CI, confidence interval; HR, hazard ratio; SD, standard deviation. [file 12944_2022_1731_MOESM3_ESM.docx]

**Supplemental Material**

**Table S1 Clinical characteristics stratified by Mean TyG index level.**

|  | **T1**  **(<8.53; n=552)** | **T2**  **(8.53-8.93; n=574)** | **T3**  **(≥ 8.93; n=568)** | **P value** |
| --- | --- | --- | --- | --- |
| **Age (y)** | 58.6±9.8 | 58.3±9.5 | 56.5±9.7 | <0.001 |
| **Sex, male** | 462 (83.7) | 448 (78.0) | 429 (75.5) | 0.003 |
| **BMI, kg/m^2^** | 25±2.9 | 26.2±3 | 26.8±3.2 | <0.001 |
| **Risk factors** |  |  |  |  |
| Hypertension | 314 (56.9) | 346 (60.3) | 365 (64.3) | 0.041 |
| Dyslipidemia | 259 (46.9) | 334 (58.2) | 365 (64.3) | <0.001 |
| Diabetes mellitus | 143 (25.9) | 197 (34.3) | 278 (48.9) | <0.001 |
| Current smoker | 148 (26.8) | 160 (27.9) | 181 (31.9) | 0.142 |
| **Medical history** |  |  |  |  |
| Prior MI | 67 (12.1) | 69 (12) | 93 (16.4) | 0.051 |
| Prior PCI | 68 (12.3) | 93 (16.2) | 100 (17.6) | 0.04 |
| Prior CABG | 6 (1.1) | 7 (1.2) | 10 (1.8) | 0.582 |
| Prior stroke | 32 (5.8) | 40 (7.0) | 32 (5.6) | 0.592 |
| PAD | 11 (2.0) | 8 (1.4) | 11 (1.9) | 0.699 |
| CKD | 4 (0.7) | 5 (0.9) | 8 (1.4) | 0.479 |
| **ACS type** |  |  |  | 0.235 |
| STEMI | 121 (21.9) | 102 (17.8) | 104 (18.3) |  |
| NSTEMI | 84 (15.2) | 78 (13.6) | 91 (16.0) |  |
| Unstable angina | 347 (62.9) | 394 (68.6) | 373 (65.7) |  |
| **Procedure characteristics** |  |  |  |  |
| Lesion vessel number | 2.0 (1.0, 3.0) | 1.0 (1.0, 2.0) | 2.0 (1.0, 3.0) | 0.667 |
| Lesion complexity, n (%) |  |  |  |  |
| Left main lesion | 54 (9.8) | 51 (8.9) | 36 (6.3) | 0.095 |
| Bifurcation lesion | 67 (12.1) | 64 (11.1) | 50 (8.8) | 0.177 |
| CTO | 87 (15.8) | 106 (18.5) | 96 (16.9) | 0.479 |
| Target vessel territory, n (%) |  |  |  |  |
| Left main | 38 (6.9) | 35 (6.1) | 23 (4.0) | 0.105 |
| LAD | 354 (64.1) | 349 (60.8) | 309 (54.4) | 0.003 |
| LCX | 139 (25.2) | 161 (28.0) | 166 (29.2) | 0.298 |
| RCA | 172 (31.2) | 181 (31.5) | 211 (37.1) | 0.057 |
| Multivessel intervention, n (%) | 124 (22.5) | 135 (23.5) | 125 (22.0) | 0.822 |
| Stent number | 2.0 (1.0, 2.0) | 1.0 (1.0, 2.0) | 1.0 (1.0, 2.0) | 0.602 |
| Mean stent diameter, mm | 3.0±0.4 | 3.0±0.4 | 3.0±0.4 | 0.056 |
| Total stent length, mm | 36.0 (22.0, 57.0) | 33.0 (23.0, 57.0) | 36.0 (23.0, 60.0) | 0.213 |
| **Laboratory results** |  |  |  |  |
| Baseline LDL-C, mg/dL | 92.0 (68.8, 122.9) | 96.1 (78.5, 119.5) | 96.5 (74.2, 124.1) | 0.130 |
| Baseline HDL-C, mg/dL | 44.8±10.6 | 42.2±9.3 | 39.5±8.5 | <0.001 |
| Baseline TC, mg/dL | 161.0±42.3 | 165.7±40.9 | 172.6±44.8 | <0.001 |
| Baseline TG, mg/dL | 92.9 (70.8, 122.1) | 122.1 (95.6, 167.1) | 174.3 (128.3, 245.8) | <0.001 |
| Baseline FPG, mg/dL | 98.9 (90.5, 112.3) | 103.9 (93.8, 119.9) | 112.0 (98.0, 144.7) | <0.001 |
| Baseline HbA1c, % | 6.0±1.0 | 6.3±1.1 | 6.8±1.5 | <0.001 |
| Baseline creatinine, μmol/L | 70.7±14.5 | 71.9±17.9 | 72.3±17.1 | 0.239 |
| Baseline uric acid, μmol/L | 342.5±82.9 | 356.6±86 | 366.8±90 | <0.001 |
| Baseline TyG index | 8.45 (8.15, 8.74) | 8.81 (8.54, 9.11) | 9.26 (8.92, 9.64) | <0.001 |
| Mean follow-up TyG index | 8.28 (8.10, 8.41) | 8.73 (8.62, 8.81) | 9.05 (9.20, 9.45) | <0.001 |
| TyG index-SD | 0.18 (0.12, 0.26) | 0.29 (0.11, 0.27) | 0.23 (0.14, 0.34) | <0.001 |
| LVEF, % | 61.7±7.6 | 61.4±7.8 | 61±8.1 | 0.343 |
| **Medications at discharge** |  |  |  |  |
| Aspirin | 552 (100) | 574 (100) | 568 (100) | NA |
| Ticagrelor | 552 (100) | 574 (100) | 568 (100) | NA |
| Statin | 546 (98.9) | 567 (98.8) | 561 (98.8) | 0.969 |
| Ezetimibe | 104 (18.8) | 81 (14.1) | 105 (18.5) | 0.062 |
| β-Blocker | 290 (52.5) | 309 (53.8) | 347 (61.1) | 0.008 |
| ACEI/ARB | 284 (51.4) | 289 (50.3) | 308 (54.2) | 0.402 |
| Calcium-channel antagonist | 131 (23.7) | 141 (24.6) | 158 (27.8) | 0.25 |
| Oral hypoglycemic agents | 83 (15.0) | 116 (20.2) | 180 (31.7) | <0.001 |
| Metformin | 36 (6.5) | 53 (9.2) | 92 (16.2) | <0.001 |
| Alpha‑glucosidase inhibitor | 48 (8.7) | 66 (11.5) | 108 (19.0) | <0.001 |
| Meglitinide | 19 (3.4) | 24 (4.2) | 40 (7.0) | 0.013 |
| Sulfonylurea | 17 (3.1) | 23 (4.0) | 56 (9.9) | <0.001 |
| Thiazolidinediones | 3 (0.5) | 8 (1.4) | 3 (0.5) | 0.182 |
| DPP-4 inhibitor | 2 (0.4) | 4 (0.7) | 3 (0.5) | 0.742 |
| SGLT-2 inhibitors | 5 (0.9) | 0 (0) | 0 (0) | 0.006 |
| GLP-1 receptor agonist | 1 (0.2) | 0 (0) | 2 (0.4) | 0.367 |
| Insulin | 18 (3.3) | 28 (4.9) | 54 (9.5) | <0.001 |

ACEI/ARB, angiotensin converting enzyme inhibitors/angiotensin receptor blockers; BMI, body mass index; CABG, coronary artery bypass grafting; CKD, chronic kidney disease; CTO, chronic total occlusion; DPP-4, dipeptidyl peptidase-4; FPG, fasting plasma glucose; GLP-1, Glucagon-like Peptide-1; HbA1c, glycosylated hemoglobin; HDL-C, high density lipoprotein cholesterol; LAD, left anterior descending artery; LCX, left circumflex ; LDL-C, low density lipoprotein cholesterol; LVEF, left ventricular ejection fraction; MI, myocardial infarction; NSTEMI, No ST-segment elevation myocardial infarction; PAD, peripheral arterial disease; PCI, percutaneous coronary intervention; RCA, right coronary artery; SGLT-2, Sodium-glucose Cotransporter-2; STEMI, ST -segment elevation myocardial infarction; TC, total cholesterol; TG, triglyceride

**Table S2 Clinical characteristics stratified by TyG index variability**

|  | **T1 (< 0.15; n=545)** | **T1 (0.15-0.26; n=563)** | **T3 (≥0.26; n=586)** | **P** |
| --- | --- | --- | --- | --- |
| **Age (y)** | 58.6±9.9 | 58±9.7 | 56.8±9.5 | 0.006 |
| **Sex, male** | 432 (79.3) | 439 (78.0) | 468 (79.9) | 0.725 |
| **BMI, kg/m^2^** | 26.1±3.1 | 25.9±3.1 | 26.1±3.1 | 0.453 |
| **Risk factors** |  |  |  |  |
| Hypertension | 334 (61.3) | 337 (59.9) | 354 (60.4) | 0.887 |
| Dyslipidemia | 286 (52.5) | 318 (56.5) | 354 (60.4) | 0.027 |
| Diabetes mellitus | 162 (29.7) | 187 (33.2) | 269 (45.9) | <0.001 |
| Current smoker | 142 (26.1) | 164 (29.1) | 183 (31.2) | 0.157 |
| **Medical history** |  |  |  |  |
| Prior MI | 79 (14.5) | 66 (11.7) | 84 (14.3) | 0.312 |
| Prior PCI | 86 (15.8) | 86 (15.3) | 89 (15.2) | 0.957 |
| Prior CABG | 7 (1.3) | 9 (1.6) | 7 (1.2) | 0.824 |
| Prior stroke | 38 (7.0) | 31 (5.5) | 35 (6.0) | 0.584 |
| PAD | 11 (2.0) | 10 (1.8) | 9 (1.5) | 0.828 |
| CKD | 7 (1.3) | 9 (1.6) | 1 (0.2) | 0.038 |
| **ACS type** |  |  |  | 0.220 |
| STEMI | 115 (21.1) | 113 (20.1) | 99 (16.9) |  |
| NSTEMI | 84 (15.4) | 89 (15.8) | 80 (13.7) |  |
| Unstable angina | 346 (63.5) | 361 (64.1) | 407 (69.5) |  |
| **Procedure characteristics** |  |  |  |  |
| Lesion vessel number | 2.0 (1.0, 3.0) | 2.0 (1.0, 3.0) | 2.0 (1.0, 3.0) | 0.711 |
| Lesion complexity, n (%) |  |  |  |  |
| Left main lesion | 52 (9.5) | 37 (6.6) | 52 (8.9) | 0.169 |
| Bifurcation lesion | 52 (9.5) | 58 (10.3) | 71 (12.1) | 0.351 |
| CTO | 88 (16.1) | 90 (16.0) | 111 (18.9) | 0.325 |
| Target vessel territory, n (%) |  |  |  |  |
| Left main | 37 (6.8) | 21 (3.7) | 38 (6.5) | 0.051 |
| LAD | 332 (60.9) | 319 (56.7) | 361 (61.6) | 0.184 |
| LCX | 140 (25.7) | 161 (28.6) | 165 (28.2) | 0.506 |
| RCA | 186 (34.1) | 190 (33.7) | 188 (32.1) | 0.737 |
| Multivessel intervention, n (%) | 125 (22.9) | 110 (19.5) | 149 (25.4) | 0.057 |
| Stent number | 1.0 (1.0, 2.0) | 1.0 (1.0, 2.0) | 1.0 (2.0, 2.0) | 0.071 |
| Mean stent diameter, mm | 3±0.4 | 3±0.4 | 2.9±0.4 | 0.033 |
| Total stent length, mm | 35.0 (23.0, 54.0) | 33.0 (22.0, 58.0) | 36.0 (23.0, 62.0) | 0.062 |
| **Laboratory results** |  |  |  |  |
| Baseline LDL-C, mg/dL | 96.3 (76.4, 120.7) | 94.0 (72.0, 122.6) | 96.3 (75.3, 123.6) | 0.590 |
| Baseline HDL-C, mg/dL | 42.6±9.7 | 42.4±10.1 | 41.4±9.4 | 0.079 |
| Baseline TC, mg/dL | 166.2±40.6 | 165.9±46.1 | 167.3±42.0 | 0.854 |
| Baseline TG, mg/dL | 119.5 (89.4, 162.0) | 120.4 (85.0, 163.7) | 135.4 (95.6, 196.7) | <0.001 |
| Baseline FPG, mg/dL | 102.2 (93.2, 120.7) | 103.3 (93.1, 117.7) | 108.2 (95.6, 138.4) | <0.001 |
| Baseline HbA1c, % | 6.2±1.0 | 6.2±1.1 | 6.8±1.5 | <0.001 |
| Baseline creatinine, μmol/L | 72.0±18.3 | 72.0±16.4 | 71.1±15 | 0.599 |
| Baseline uric acid, μmol/L | 356.3±82.6 | 355.2±89 | 354.8±88.9 | 0.956 |
| Baseline TyG index | 8.77 (8.44, 9.12) | 8.75 (8.39, 9.15) | 8.98 (8.56, 9.39) | <0.001 |
| Mean follow-up TyG index | 8.66 (8.38, 8.95) | 8.68 (8.36, 8.97) | 8.87 (8.52, 9.21) | <0.001 |
| TyG index-SD | 0.10 (0.06, 0.12) | 0.20 (0.17, 0.22) | 0.33 (0.29, 0.40) | <0.001 |
| LVEF, % | 61.2±7.9 | 61.5±7.5 | 61.4±8.1 | 0.779 |
| **Medications at discharge** |  |  |  |  |
| Aspirin | 545 (100) | 563 (100) | 586 (100) | NA |
| Ticagrelor | 545 (100) | 563 (100) | 586 (100) | NA |
| Statin | 538 (98.7) | 558 (99.1) | 578 (98.6) | 0.728 |
| Ezetimibe | 96 (17.6) | 96 (17.1) | 98 (16.7) | 0.923 |
| β-Blocker | 292 (53.6) | 304 (54.0) | 350 (59.7) | 0.064 |
| ACEI/ARB | 286 (52.5) | 273 (48.5) | 322 (54.9) | 0.088 |
| Calcium-channel antagonist | 146 (26.8) | 132 (23.4) | 152 (25.9) | 0.411 |
| Oral hypoglycemic agents | 91 (16.7) | 124 (22.0) | 164 (28.0) | <0.001 |
| Metformin | 45 (8.3) | 61 (10.8) | 75 (12.8) | 0.047 |
| Alpha‑glucosidase inhibitor | 46 (8.4) | 69 (12.3) | 107 (18.3) | <0.001 |
| Meglitinide | 17 (3.1) | 29 (5.2) | 37 (6.3) | 0.043 |
| Sulfonylurea | 27 (5.0) | 28 (5.0) | 41 (7.0) | 0.227 |
| Thiazolidinediones | 2 (0.4) | 7 (1.2) | 5 (0.9) | 0.272 |
| DPP-4 inhibitor | 3 (0.6) | 4 (0.7) | 2 (0.3) | 0.689 |
| SGLT-2 inhibitors | 2 (0.4) | 1 (0.2) | 2 (0.3) | 0.818 |
| GLP-1 receptor agonist | 1 (0.2) | 1 (0.2) | 1 (0.2) | 0.999 |
| Insulin | 22 (4.0) | 27 (4.8) | 51 (8.7) | 0.002 |

ACEI/ARB, angiotensin converting enzyme inhibitors/angiotensin receptor blockers; BMI, body mass index; CABG, coronary artery bypass grafting; CKD, chronic kidney disease; CTO, chronic total occlusion; DPP-4, dipeptidyl peptidase-4; FPG, fasting plasma glucose; GLP-1, Glucagon-like Peptide-1; HbA1c, glycosylated hemoglobin; HDL-C, high density lipoprotein cholesterol; LAD, left anterior descending artery; LCX, left circumflex ; LDL-C, low density lipoprotein cholesterol; LVEF, left ventricular ejection fraction; MI, myocardial infarction; NSTEMI, No ST-segment elevation myocardial infarction; PAD, peripheral arterial disease; PCI, percutaneous coronary intervention; RCA, right coronary artery; SGLT-2, Sodium-glucose Cotransporter-2; STEMI, ST -segment elevation myocardial infarction; TC, total cholesterol; TG, triglyceride

**Table S3 The risk of endpoints based on TyG index level and variability.**

|  | **Mean TyG index** | |  | **TyG index -SD** | |
| --- | --- | --- | --- | --- | --- |
|  | HR (95% CI) | **P value** |  | HR (95% CI) | **P value** |
| **MACCE** | | | | | |
| T1 | Reference | — |  | Reference | — |
| T2 | 1.59 (0.88, 2.86) | 0.125 |  | 1.51 (0.86, 2.65) | 0.152 |
| T3 | 2.73 (1.57, 4.75) | <0.001 |  | 2.17 (1.28, 3.68) | 0.004 |
| **All-cause death** | | | | | |
| T1 | Reference | — |  | Reference | — |
| T2 | 1.16 (0.16, 8.27) | 0.885 |  | 4.41 (0.51, 37.95) | 0.176 |
| T3 | 2.16 (0.35, 13.16) | 0.405 |  | 0.78 (0.05, 12.74) | 0.864 |
| **Non-fatal MI** | | | | | |
| T1 | Reference | — |  | Reference | — |
| T2 | 1.17 (0.31, 4.4) | 0.813 |  | 4.97 (0.58, 42.61) | 0.143 |
| T3 | 1.63 (0.46, 5.76) | 0.450 |  | 8.74 (1.11, 68.59) | 0.039 |
| **Ischemic stroke** | | | | | |
| T1 | Reference | — |  | Reference | — |
| T2 | 1.51 (0.11, 20.14) | 0.755 |  | 0.18 (0.02, 1.55) | 0.117 |
| T3 | 0.87 (0.06, 12.54) | 0.917 |  | 4.04 (0.45, 36.3) | 0.213 |
| **Unplanned repeat revascularization** | | | | | |
| T1 | Reference | — |  | Reference | — |
| T2 | 1.51 (0.79, 2.9) | 0.216 |  | 1.32 (0.71, 2.43) | 0.377 |
| T3 | 2.97 (1.64, 5.38) | <0.001 |  | 1.86 (1.06, 3.27) | 0.032 |

CI confidence interval; CV, coefficient of variation; HR, hazard ratio; MACCE, major advent cardiovascular and cerebrovascular event; MI, myocardial infarction; SD, standard deviation;

adjusted for age, sex, hypertension, dyslipidemia, diabetes mellitus, prior MI, Prior PCI, Prior CABG, PAD, mean stent diameter, β-blocker, oral hypoglycemic agents, insulin, baseline LDL-C, baseline TC, baseline FBG, baseline TyG index and baseline HbA1C

Mean TyG index: T1<8.53 vs T2 8.53-8.93 vs T3 ≥8.93

TyG index -SD: T1<0.15 vs T2 0.15-0.26 vs T3≥0.26


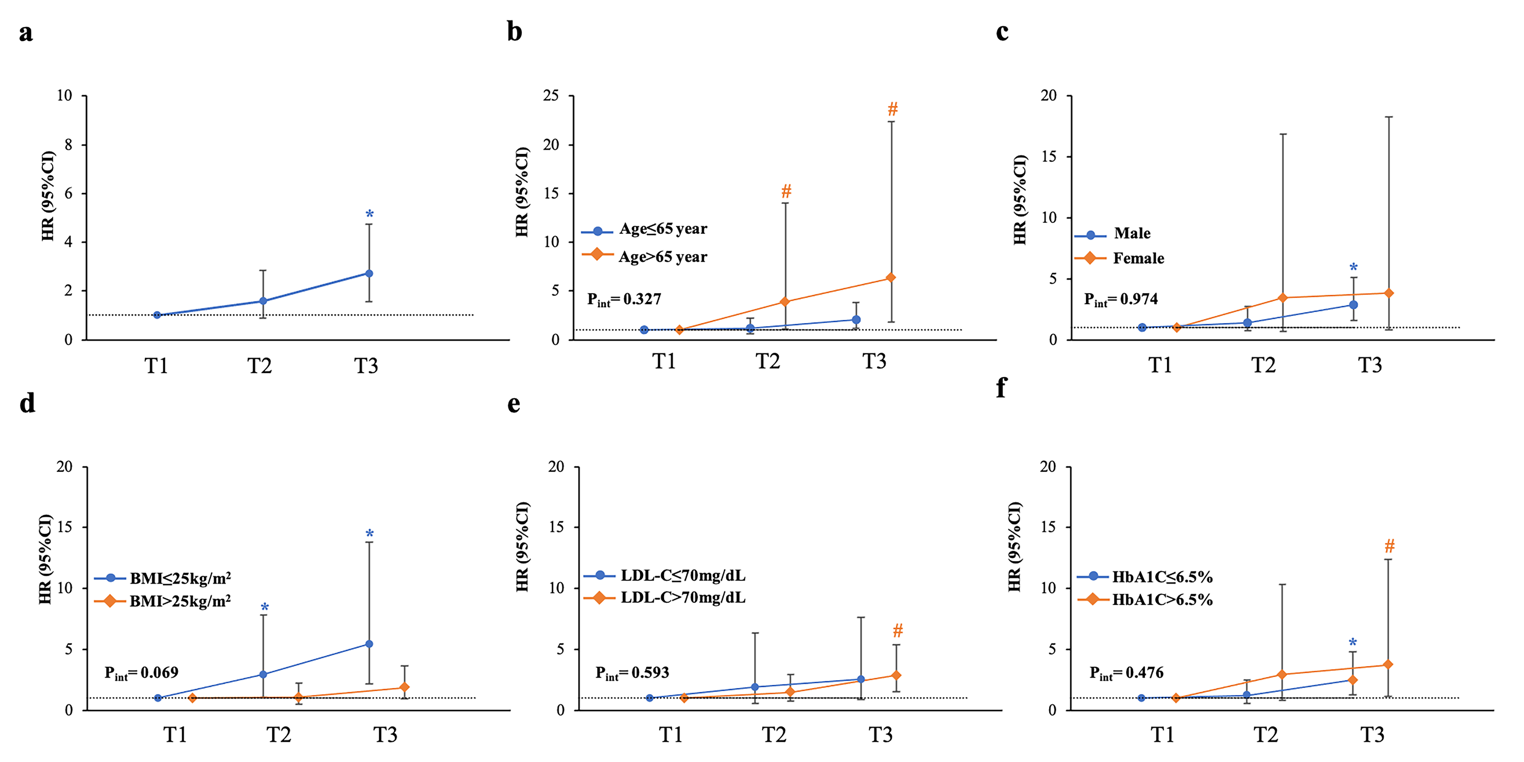


**Figure S1.** Association of TyG index level and major adverse cardiovascular events across subgroups. Hazard ratios for major adverse cardiovascular events by tertiles of mean TyG index in the overall population (a) and across subgroups of age (b), sex (c), BMI (d), LDL-C (e), and HbA1c (f). BMI, body mass index; HbA1c, hemoglobin A1c; LDL-C, low-density lipoprotein cholesterol. CI, confidence interval; HR, hazard ratio.


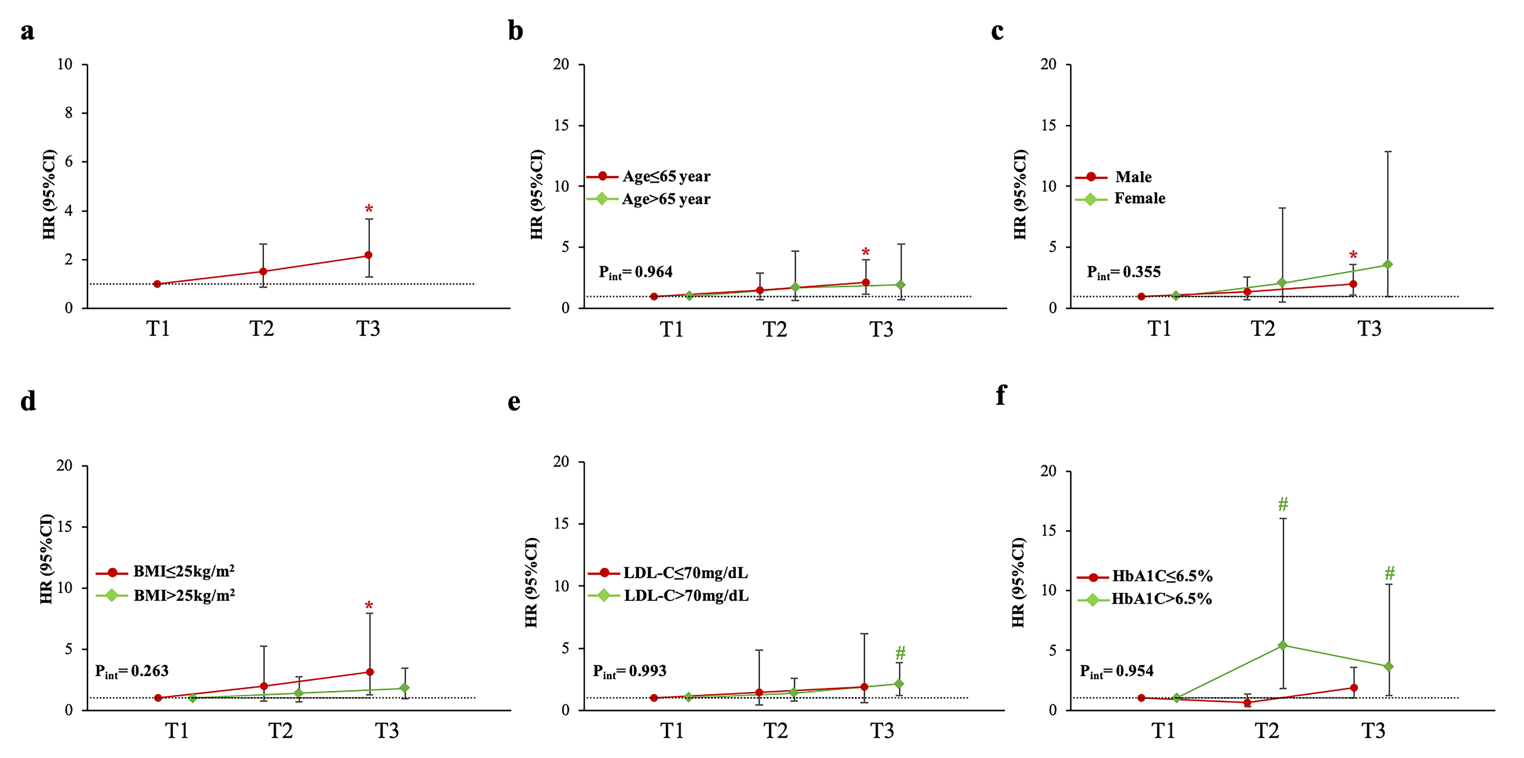


**Figure S2.** Association of TyG index variability and major adverse cardiovascular events across subgroups. Hazard ratios for major adverse cardiovascular events by tertiles of TyG index-SD in the overall population (a) and across subgroups of age (b), sex (c), BMI (d), LDL-C (e), and HbA1c (f). BMI, body mass index; HbA1c, hemoglobin A1c; LDL-C, low-density lipoprotein cholesterol. CI, confidence interval; HR, hazard ratio; SD, standard deviation.
